# Wavelength and Fibrosis Affect Phase Singularity Locations During Atrial Fibrillation

**DOI:** 10.3389/fphys.2018.01207

**Published:** 2018-09-10

**Authors:** Mirabeau Saha, Caroline H. Roney, Jason D. Bayer, Marianna Meo, Hubert Cochet, Remi Dubois, Edward J. Vigmond

**Affiliations:** ^1^IMB, UMR 5251, University of Bordeaux, Pessac, France; ^2^IHU Liryc, Electrophysiology and Heart Modeling Institute, Fondation Bordeaux University, Pessac, France; ^3^Department of Biomedical Engineering, King's College London, London, United Kingdom

**Keywords:** atrial fibrillation, computer simulation, phase singularity mapping, Fibrosis, repolarization heterogeneity

## Abstract

The mechanisms underlying atrial fibrillation (AF), the most common sustained cardiac rhythm disturbance, remain elusive. Atrial fibrosis plays an important role in the development of AF and rotor dynamics. Both electrical wavelength (WL) and the degree of atrial fibrosis change as AF progresses. However, their combined effect on rotor core location remains unknown. The aim of this study was to analyze the effects of WL change on rotor core location in both fibrotic and non-fibrotic atria. Three patient specific fibrosis distributions (total fibrosis content: 16.6, 22.8, and 19.2%) obtained from clinical imaging data of persistent AF patients were incorporated in a bilayer atrial computational model. Fibrotic effects were modeled as myocyte-fibroblast coupling + conductivity remodeling; structural remodeling; ionic current changes + conductivity remodeling; and combinations of these methods. To change WL, action potential duration (APD) was varied from 120 to 240ms, representing the range of clinically observed AF cycle length, by modifying the inward rectifier potassium current (*I*_*K*1_) conductance between 80 and 140% of the original value. Phase singularities (PSs) were computed to identify rotor core locations. Our results show that *I*_*K*1_ conductance variation resulted in a decrease of APD and WL across the atria. For large WL in the absence of fibrosis, PSs anchored to regions with high APD gradient at the center of the left atrium (LA) anterior wall and near the junctions of the inferior pulmonary veins (PVs) with the LA. Decreasing the WL induced more PSs, whose distribution became less clustered. With fibrosis, PS locations depended on the fibrosis distribution and the fibrosis implementation method. The proportion of PSs in fibrotic areas and along the borders varied with both WL and fibrosis modeling method: for patient one, this was 4.2–14.9% as *I*_*K*1_ varied for the structural remodeling representation, but 12.3–88.4% using the combination of structural remodeling with myocyte-fibroblast coupling. The degree and distribution of fibrosis and the choice of implementation technique had a larger effect on PS locations than the WL variation. Thus, distinguishing the fibrotic mechanisms present in a patient is important for interpreting clinical fibrosis maps to create personalized models.

## 1. Introduction

Cardiac arrhythmia is a major cause of death, with considerable health and economic impacts. Atrial Fibrillation (AF) is the most common cardiac arrhythmia. It leads to heart failure and stroke (Kannel et al., 1998). The understanding of the pathophysiology of AF remains incomplete despite numerous experimental and clinical studies, as well as several *in-silico* studies (Wolf et al., 1991; Courtemanche et al., 1998; Labarthe et al., 2014). Currently, because of its easy reproducibility, its inexpensiveness and its non-invasiveness, computational modeling is widely used to investigate the mechanisms underlying AF.

The underlying atrial substrate is essential for AF maintenance (Kostin et al., 2002). For many patients, the success of anti-arrhythmic drug therapy is low. Thus, catheter ablation is used to eliminate arrhythmogenic electrical sources by destroying the underlying tissue substrate (for example by isolating the pulmonary veins or by targeting potential reentrant sources) using localized energy delivery (Earley et al., 2006). However, AF is recurrent in 30–50% of patients (Calkins et al., 2012). The sub-optimal success rate of AF treatment by catheter ablation procedures is confounded by a lack of consensus between clinicians on the precise locations to be targeted (Schotten et al., 2011; Calkins et al., 2012).

AF dynamics are strongly influenced by heterogeneities in the electrophysiological tissue properties (Kottkamp et al., 2016) and the spatial pattern of tissue structural changes, which are patient specific. Cardiac wavelength (WL)—the distance traveled by the depolarization wave during the functional refractory period (Jacquemet et al., 2005)—is shortened by structural and electrical remodeling in tissue with chronic or persistent AF (Krogh-Madsen et al., 2012). Previous studies have shown that modifying WL properties (through action potential duration, APD, variation) affects AF dynamics (Qu et al., 1999). However, Deng et al. (2017) found that with modest changes in electrophysiological properties, reentrant drivers localized to the same areas, at the edges of high fibrotic regions.

Furthermore, myocardial fibrosis affects AF maintenance (Morgan et al., 2016; Cochet et al., 2018). Fibrotic remodeling is multi-factorial, and includes interstitial fibrosis (the deposition of collagen between fiber bundles), replacement fibrosis, gap junctional remodeling due to downregulation of connexin 43, fibroblast coupling and paracrine factors. These factors may initiate and sustain AF by modifying conduction pathways. Previous studies have suggested targeting fibrotic areas during catheter ablation procedures.

In this study, we investigate how WL affects AF driver location using a computational bilayer model of the human atria, incorporating fibrosis distributions based on late gadolinium enhancement (LGE) Magnetic Resonance Imaging (MRI) scans from patients with persistent AF. In comparison with previous studies, we explore a larger range of clinically observed APD, consider multiple techniques for modeling atrial fibrosis, and directly compare with clinical rotor core trajectory data.

## 2. Methods

### 2.1. AF bilayer model

We used our previously described human biatrial biophysically-detailed bilayer model (Labarthe et al., 2014), with AF simulated as in our former work (Bayer et al., 2016). Briefly, the bilayer model is a finite element model composed of discretely connected 2D layers. The right atrium (RA) consists of an epicardial layer and a layer for the crista terminalis (CT) and the pectinate muscles (PM). The left atrium (LA) comprises endocardial and epicardial layers. Each node on the LA endocardium and RA endocardial structures (the CT and PM) is connected by a discrete resistance to the closest node on the LA or RA epicardial surface, respectively. Interatrial connections include Bachmann's bundle, the rim of the fossa ovalis, and the muscular sheath of the coronary sinus. The mesh contains 363561 nodes with an edge length of approximately 300μ*m*. Atrial action potentials (AP) vary from region to region (Gelband et al., 1972). As in previous work (Bayer et al., 2016; Roney et al., 2018), the Courtemanche-Ramirez-Nattel human atrial ionic model (Courtemanche et al., 1998) was used and ionic channel conductances varied to recreate the observed heterogeneity (Aslanidi et al., 2011; Krueger et al., 2013). This ionic model is sufficient since we are mainly concerned with APD restitution, and not the detailed subcellular processes seen in newer models which carry significantly increased computational costs. We also incorporate heterogeneous coupling, resulting in different conduction velocities (CVs) in different anatomical regions, according to human data (Lemery et al., 2007; Bayer et al., 2016). Furthermore, to simulate chronic AF, the following electrical remodeling was incorporated: a 50% decrease in the conductance of the atrial-specific ultra-rapid potassium current *I*_*Kur*_, a 50% decrease in the conductance of the transient outward current *I*_*to*_, and a 70% decrease in the conductance of the L-type calcium current *I*_*CaL*_ (Courtemanche et al., 1999).

### 2.2. Wavelength variation

WL is the product of the effective refractory period (ERP) and CV (Jacquemet et al., 2005), which we approximate as APD × CV. Changing the conductance of *I*_*K*1_ alone does not affect CV (Entz and William, 2018; Nobles et al., 2018; Valli et al., 2018). Pandit and Jalife (2013) found that the inward rectifier *K*^+^ channel (*I*_*K*1_) has an important role on the spatiotemporal organization of AF drivers across different parts of the heart. Specifically, changes in *I*_*K*1_ conductance lead to variation of rotor (AF driver) frequency and tip meander because of changes in APD and in the resting level of the membrane potential (which in turn affects cardiac excitability). Previous studies have investigated the effects of *I*_*K*1_ conductance on APD (Dhamoon and Jalife, 2005; Pandit et al., 2005; Lee et al., 2016). We multiplied *I*_*K*1_ conductance by 80, 95, 100, 120, and 140 to modify the APD, and thereby the WL. These values were chosen to give tissue AF cycle lengths in the clinically reported range of 120–240 ms (Konings et al., 1994; Ng et al., 2006).

### 2.3. Patient fibrosis data and fibrosis modeling

#### 2.3.1. Patient data

**Figure 1:**
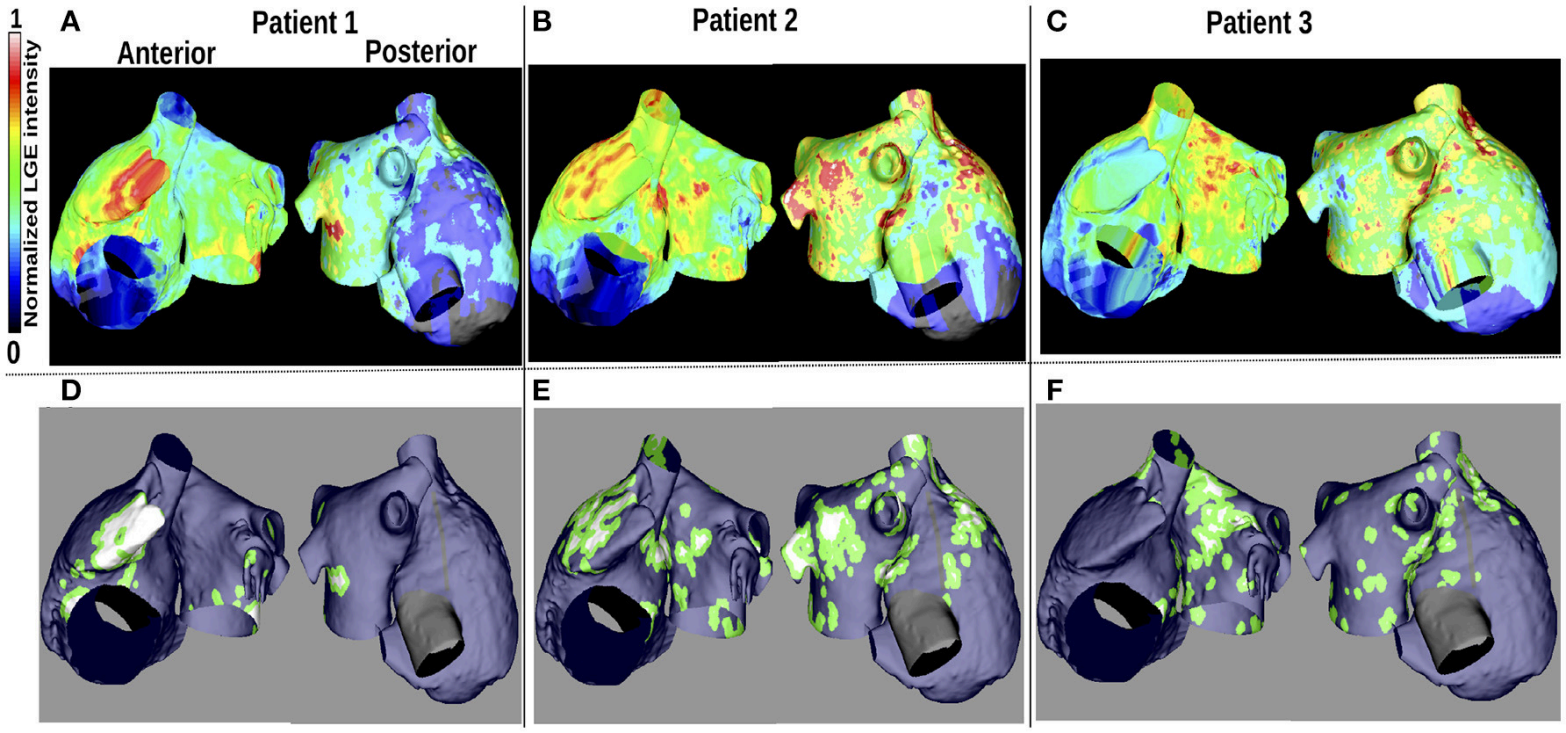
Patient LGE data and fibrosis distribution used for modeling. **(A–C)**: Normalized LGE intensity. **(D–F)**: Fibrotic tissue (white), non-fibrotic areas (blue), and the border between them (green) are shown. The inferior vena cava and line of block regions of the model are electrically inactive and displayed in black in **(D–F)**.

LGE-MRI intensity data from three patients with persistent AF (Cochet et al., 2015) were projected onto the aforementioned computational atrial bilayer model to assign a fibrosis distribution. For each patient, these intensity values were assigned for each point on an endocardial surface mesh as the maximum value through the atrial wall. These distributions of LGE intensity values were then mapped to the atrial bilayer model using an iterative closest point algorithm (Roney et al., 2016; Cochet et al., 2018). Each of the patients gave informed written consent. The ethics committee of the University of Bordeaux approved the study and it complies with the declaration of Helsinki. Figures 1A–C show the distribution maps of LGE intensity, normalized with respect to the highest value for each patient. We considered an identical LGE intensity distribution on the LA epicardium and endocardium. For the RA, we included atrial fibrosis in the epicardial layer of the model, but not on the endocardial structures. To incorporate atrial fibrosis in the models, nodes with normalized LGE intensity greater than 0.7 were defined to be fibrotic. This corresponds to 13.2, 27.2, and 27.5% of the LA tissue being classified as fibrotic, for patients 1, 2, and 3 respectively (16.6, 22.8, and 19.2% of the entire atria). Since previous studies have found phase singularities (PSs) on the border between fibrosis and normal myocardium (Zahid et al., 2016; Cochet et al., 2018), we defined nodes within 0.75*mm* of the boundary to be in the border region (Figures 1A–F). This distance, representing a region about five elements wide, allowed us to be specific, while respecting clinical resolution. This width is less than an ablation line, which is usually greater than 2 mm (Bhaskaran et al., 2016). Therefore, we ensure that if the ablation strategy follows such a border, the entire width of the border would be ablated. This border region is only considered for post-processing analyses and does not affect the model construction. The border region corresponds to 38.2, 62.4, and 71.9% of the total fibrotic area (normalized LGE intensity greater than 0.7) for patient 1–3, respectively.

#### 2.3.2. Fibrosis implementation methods

Computational modeling studies use different methodologies to model fibrosis (Morgan et al., 2016; Vigmond et al., 2016; Zahid et al., 2016), and previous studies show that the reentry dynamics are affected by the fibrosis implementation method. To study how rotor core position changes when WL is varied in fibrotic atria, we used three different implementation methods: structural remodeling, ionic remodeling and fibroblast coupling, as well as two combinations: structural + ionic changes, and structural changes + fibroblast coupling.

#### Structural remodeling

Vigmond et al. (2016) and Roney et al. (2016) give detailed descriptions of this method. It consists of randomly removing elements from the mesh, with a probability depending on the normalized LGE intensity.

#### Ionic remodeling

To incorporate ionic remodeling, we rescaled the ionic conductances of the human atrial AF ionic cell model, following Zahid et al. (2016). Specifically, in fibrotic regions, ionic conductances were rescaled as follows: 50% reduction in inward rectifier potassium current (*I*_*K*1_), 50% reduction in L-type calcium current (*I*_*CaL*_), and 40% reduction in the sodium current (*I*_*Na*_). To take into account the impact of fibrosis on the intermyocyte coupling, the longitudinal conductivity in the fibrotic regions of the model was reduced by 30%, and then the longitudinal-to-transverse anisotropy ratio was increased to 8:1 to model additional slowing of velocity in the transverse direction.

#### Fibroblast coupling

We used the Morgan et al. (2016) model of atrial fibroblasts. Briefly, the interaction between fibroblast and myocyte cells is modeled with a gap junctional conductance of 0.5*nS*. For each patient, the normalized LGE intensity was divided into six intervals (0−0.27−0.4−0.55−0.69−0.82−1). These intervals correspond to one non-fibrotic region and five different degrees of fibrotic remodeling, ranging from diffuse (1 and 2 fibroblasts per myocyte) to patchy (3 and 4 fibroblasts per myocyte) and dense (5 fibroblasts per myocyte). The myocyte-myocyte longitudinal coupling conductivity was decreased from 100% (in regions with no fibroblast coupling) to 20% of the baseline value (in regions with coupling to 5 fibroblasts per myocyte), following Morgan et al. (2016).

### 2.4. Simulation protocol

We used the monodomain formulation and solved it with the Cardiac Arrhythmia Research Package (CARP) simulator (Vigmond et al., 2003). For each case, we first simulated the model for 2 seconds without electrical stimulation, followed by 3.5 s of sinus rhythm with a cycle length of 0.7 seconds (Roney et al., 2016). Five rapid pacing pulses, with a cycle length in the range 112–184 ms, depending on model inducibility, were applied to the upper right pulmonary vein (URPV) to initiate AF. We simulated each model for 2.1 s to allow AF arrhythmia dynamics to stabilize and the final 2.1 seconds were used for analysis for a total simulation time of 9.7 s. All models were inducible.

### 2.5. Analysis of the effects of wavelength variations on PS positions

To estimate WL, action potential duration at 90% repolarization (APD90) and CV were calculated over the atrial mesh for each simulation set-up during sinus rhythm. Local CV was computed as the magnitude of conduction velocity. WL was then estimated as the product of APD90 and CV for each node of the mesh.

PSs were located by the intersection of the isopotential line *V*_*m*_ = −20 mV and the line of *dV*_*m*_/*dt* = 0 (Bishop and Plank, 2012). We plotted and analyzed changes in the percentage (defined as the number of PSs in an area over the total number of PSs in the model) and number of PSs per time unit (each ms) located in each atrial region as *I*_*K*1_ was varied. We analyzed the following atrial regions separately: the PVs [the upper left pulmonary vein (ULPV), the upper right pulmonary vein (URPV), the left lower pulmonary vein (LLPV) and the lower right pulmonary vein (LRPV)], the fossa-ovalis (FO), the superior vena cava (SVC), the LA and the RA. In models with fibrosis, we analyzed the PS content of the fibrotic areas and their borders. To reduce the computation time, parameters were evaluated on a reduced mesh, excluding the electrically inactive regions (the inferior vena cava and the septal line of block), Bachmann's bundle, the crista terminalis, and the pectinate muscles.

## 3. Results

### 3.1. Physiological changes due to I_*K*1_ variation

**Figure 2:**
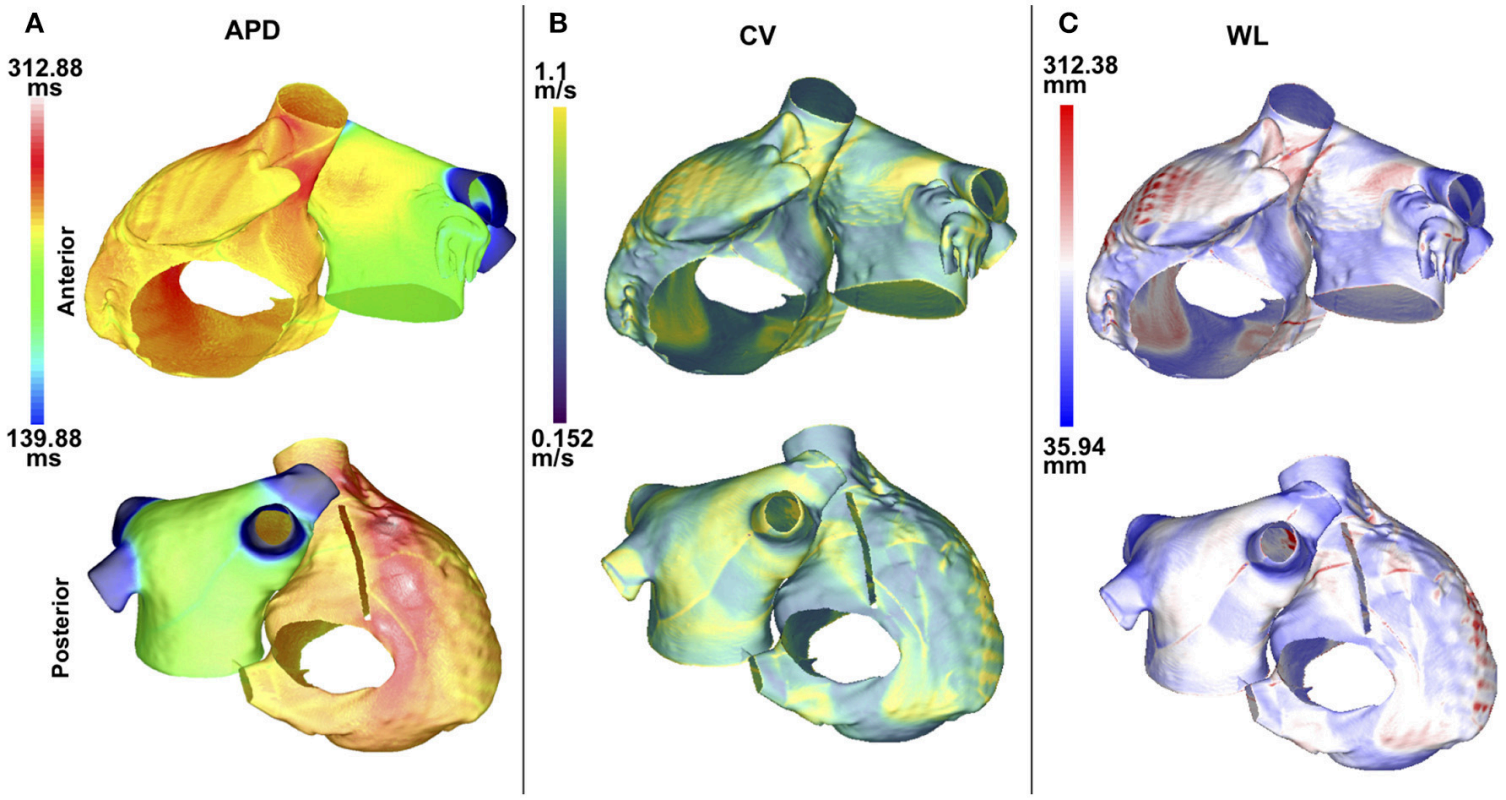
APD, CV, and WL maps are spatially heterogeneous in the control case without fibrosis. Anteroposterior (top row) and posteroanterior (bottom row) views are shown. **(A)** APD90 map. **(B)** CV map. **(C)** WL map. Data are displayed on a reduced mesh for 80*%I*_*K*1_.

APD90, CV, and WL were computed for 5 values of *I*_*K*1_ conductance. For each of these measurements, the maximum values were located in the same atrial region for all of the *I*_*K*1_ values; this was also the case for the minimum values. Maps of these quantities are illustrated in Figure 2. The maximum and minimum APD90 changed when *I*_*K*1_ was varied, as shown in Table 1. As *I*_*K*1_ conductance was increased from 80% to 140% of the control value, *APD*_*min*_ decreased from 139.9 to 120.5 ms and *APD*_*max*_ from 312.9 to 227.1 ms, respectively. The maximum and minimum CV were ~1.1 and ~0.15 m/s, respectively. The CV extrema exhibit little variation as *I*_*K*1_ increased from 80% to 140% of control. The minimum and maximum WL decreased from 35.9 to 28.4 mm, and from 312.4 to 225.6 mm, respectively, when *I*_*K*1_ varied from 80 to 140% of control as shown in Table 1. These WL calculations are local and based on nonuniform propagation; APD and CV are influenced by wavefront curvature, changes in fiber direction, propagation into inexcitable borders, and wavefront collision.

**Table 1:** Physiological changes due to *I*_*K*1_ variation.

***I*_*K*1_ percentage**	**80%**	**95%**	**100%**	**120%**	**140%**
APD90min (ms)	139.9	134.7	133.1	127.3	120.5
APD90max (ms)	312.9	285.5	269.1	245.1	227.1
WLmin (mm)	35.9	30.1	28.2	29.3	28.4
WLmax (mm)	312.4	281.7	274.2	246.0	225.6

*APDmin and APDmax are minimum and maximum APD, respectively. WLmin and WLmax are minimum and maximum WL, respectively*.

The APD90 map is almost uniform on the LA posterior wall. On the LA anterior wall, the APD is higher around Bachmann's bundle than on the rest of the wall. This results in a large gradient of APD around the connection area. The CV and WL are higher on the LA wall area close to the LAA and inferior PV intersections. The highest APD, CV and WL values are seen on the RA posterior wall at the cristae terminalis and pectinate muscles, and near the anterior SVC junction. There are gradients in APD and WL around the intersections of the LA wall with the PVs.

### 3.2. PS location with no fibrosis

**Figure 3:**
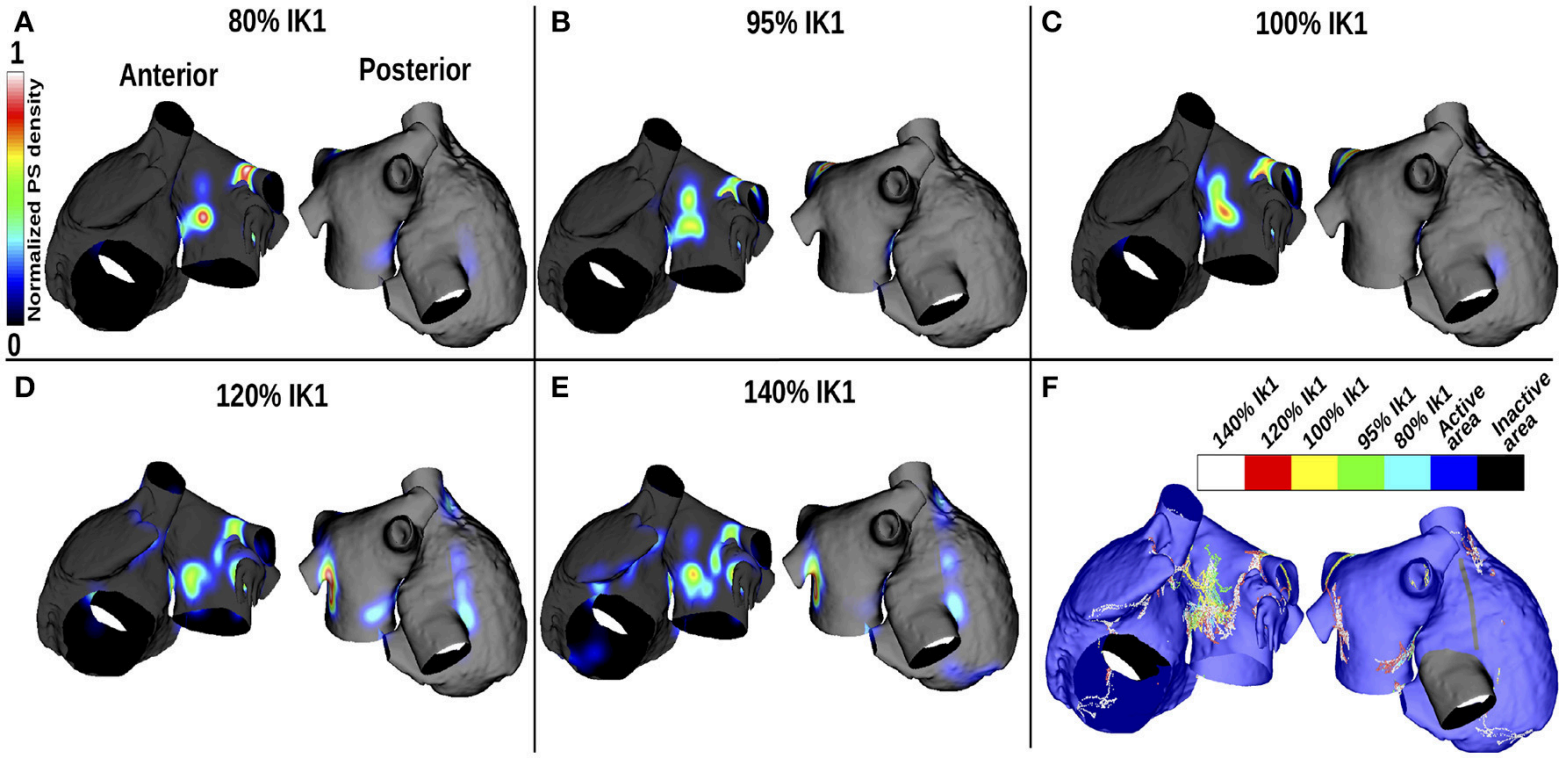
PS density maps change with APD variation for the control case of no atrial fibrosis. Each subfigure shows anteroposterior (left) and posteroanterior (right) views. **(A–E)** display PS density distributions for 5 *I*_*K*1_ values. **(F)** Shows PS positions displayed on the same mesh for the different *I*_*K*1_ values.

Figures 3A–E show changes in PS density as *I*_*K*1_ is varied, and Figure 3F shows a corresponding map indicating the PS locations. For small *I*_*K*1_ values (corresponding to large APD values), rotor organizing centers were more likely to appear on the LA than the RA, in the center of the anterior wall and around the lower PVs intersections, where the APD gradients are high (see Figure 2). As the WL decreased (corresponding to increased *I*_*K*1_), the PS density became less clustered and more widespread over the LA. In addition, PS trajectories covered larger areas of the RA. Concurrently, in the LA, the PS density decreased progressively as WL decreased, and more PS positions appeared.

**Figure 4:**
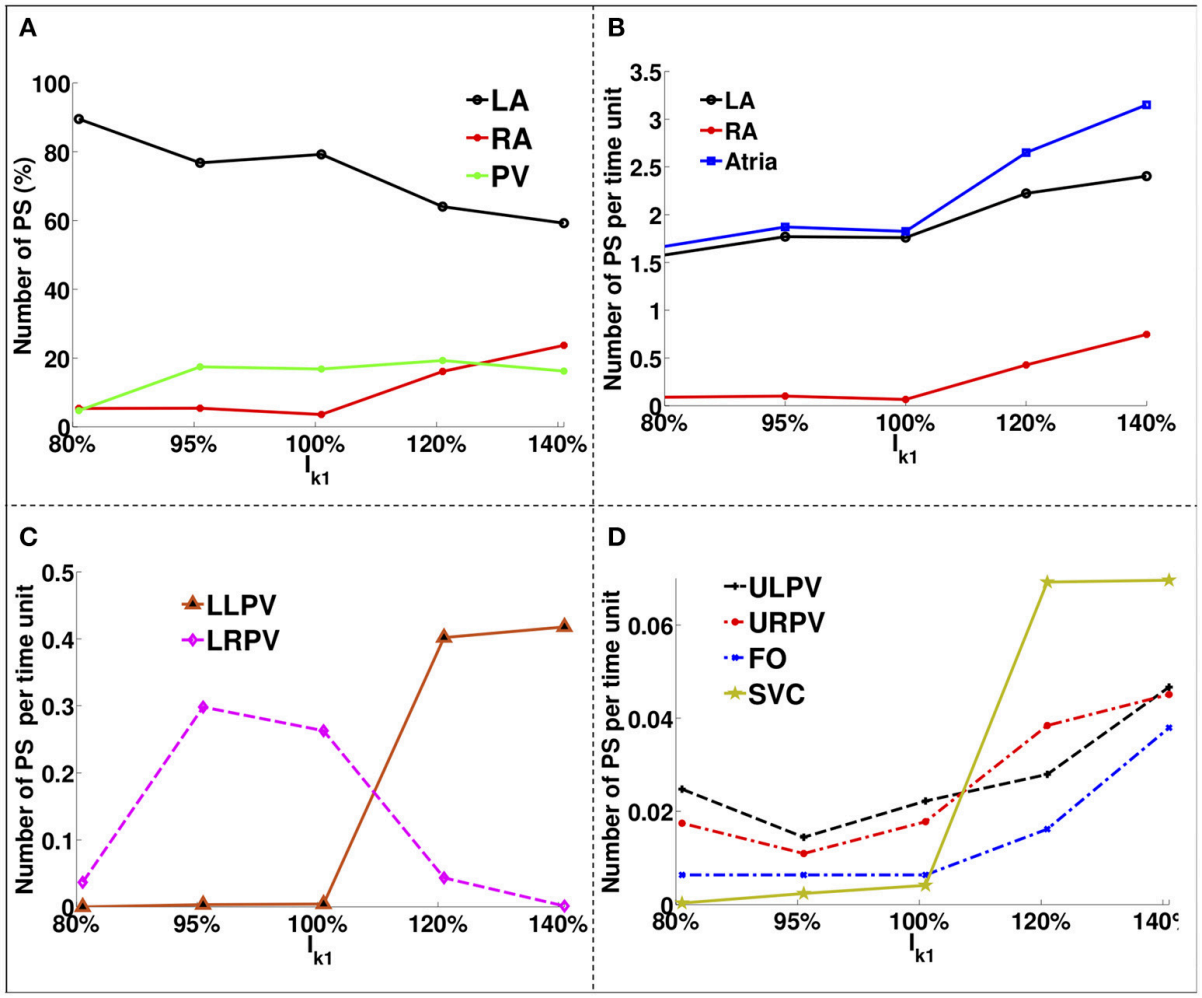
PS location variation as a function of *I*_*K*1_ for atria with no fibrosis. **(A)** Percentage of PS locations on the LA body, on the RA and on the PVs. **(B)** Number of PSs per unit time on the entire LA, on the RA and on the whole atria. **(C)** Number of PSs per time unit on the LLPV and on the LRPV. **(D)** Number of PSs per unit time on the ULPV, URPV, FO, and SVC.

Figure 4 shows how the number and locations of PSs change in different areas as the *I*_*K*1_ conductance was varied. The percentage of PS positions on the LA decreases from ~90% for large APD values to ~60% for short APD values, while it increases in the RA and PVs from ~5% each to ~22% and ~18%, respectively. The number of PSs increases with *I*_*K*1_ conductance for all regions. There were a greater number of PSs on the LA than on the RA for all *I*_*K*1_ conductance values.

The LA had ~1.6 PS positions per time unit compared to ~0.1 in the RA for large APD, while for short APD, the LA had ~2.3 compared to ~0.9 in the RA. The lower PVs (Figure 4C) had an order of magnitude more PSs than the upper PVs (Figure 4D). The number of PSs on the FO and SVC increased monotonically with *I*_*K*1_ conductance. For large APD values, there were no PS located on the LLPV or on the SVC, but as APD was decreased, the number of PS on both the LLPV and SVC increased.

### 3.3. PS location analyses for cases with fibrosis

Rotor core location depends on both fibrosis distribution and WL. Results are presented in full for one patient; similar results for the other patients are presented in the Supplementary Material.

**Figure 5:**
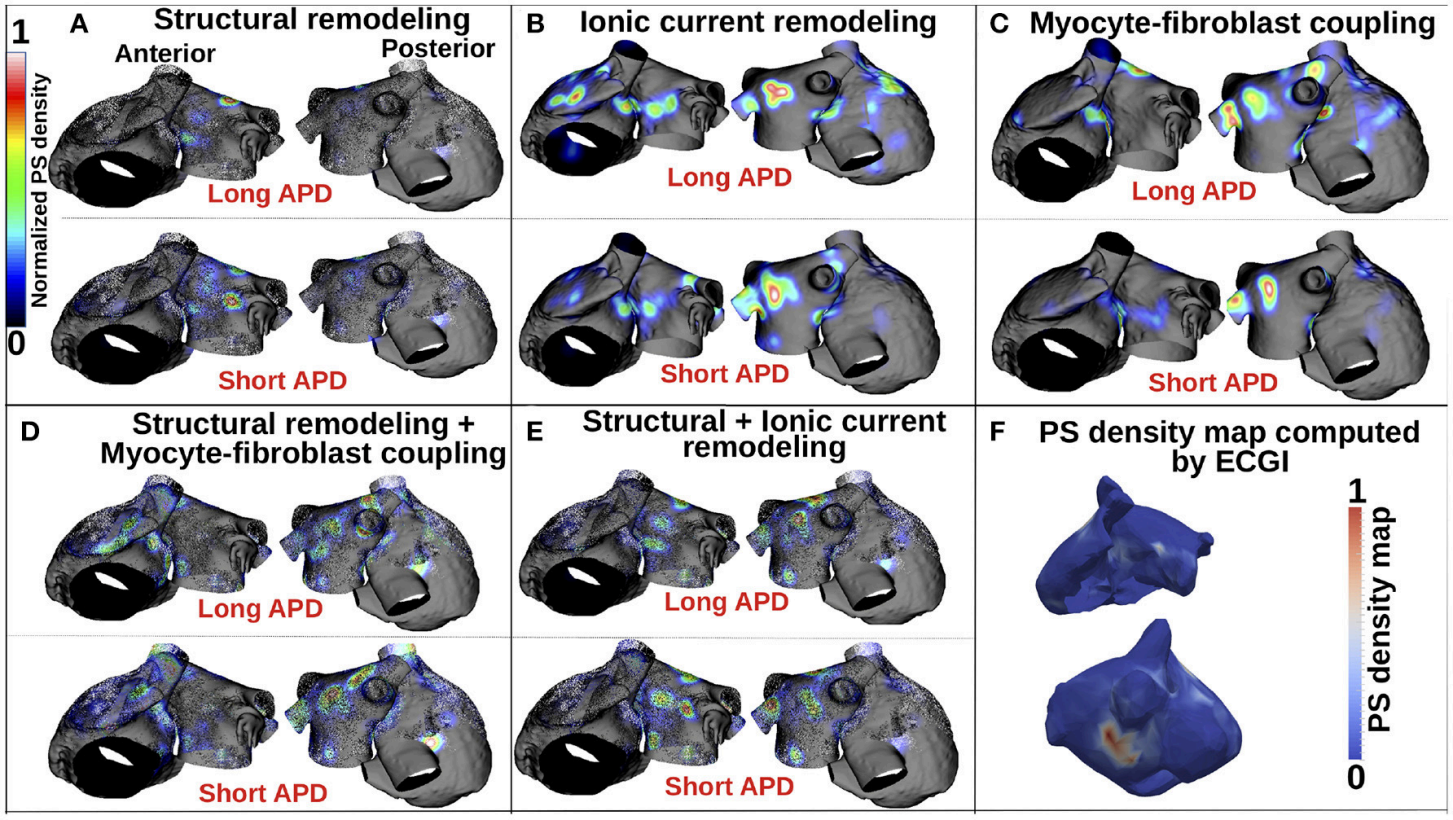
PS density maps depend on both fibrosis implementation method and APD. **(A–E)** Show PS density maps for the 5 fibrosis implementation methods: structural remodeling, ionic current remodeling, myocyte-fibroblast coupling, structural remodeling + myocyte-fibroblast coupling, and structural + ionic current remodeling, respectively. **(F)** Clinical PS density map computed using electrocardiographic imaging (ECGi). These data are for patient 2; see Supplementary Figure 1 for patients 1 and 3.

Figure 5 presents PS location maps for patient 2 as *I*_*K*1_ conductance was varied from 80 to 140% for the five fibrosis implementation methods: structural changes, ionic current remodeling, myocyte-fibroblast coupling, the combination of structural + ionic current remodeling, and the combination of structural changes + myocyte-fibroblast coupling. An area of moderate PS density on the anterior wall of the LA and RA is seen in the clinical data (Figure 5F), and also in each of the simulations. This area of agreement is present for all of the fibrosis implementations. There is an area of high PS density on the posterior wall of the LA in the clinical, as presented in Figure 5F, that is not reproduced in the simulation results.

Supplementary Figure 1 presents PS density maps corresponding to long and short APD values for each of the fibrosis modeling methodologies, for each of the patients. In general, there is a good visual correspondence between PS density maps for long and short APDs, and a much larger variation in PS density maps between fibrosis implementation methods. The correlation between PS density maps as APD is varied is greatest for the ionic method (see Supplementary Tables 1–3), and generally lower for the structural remodeling method.

**Figure 6:**
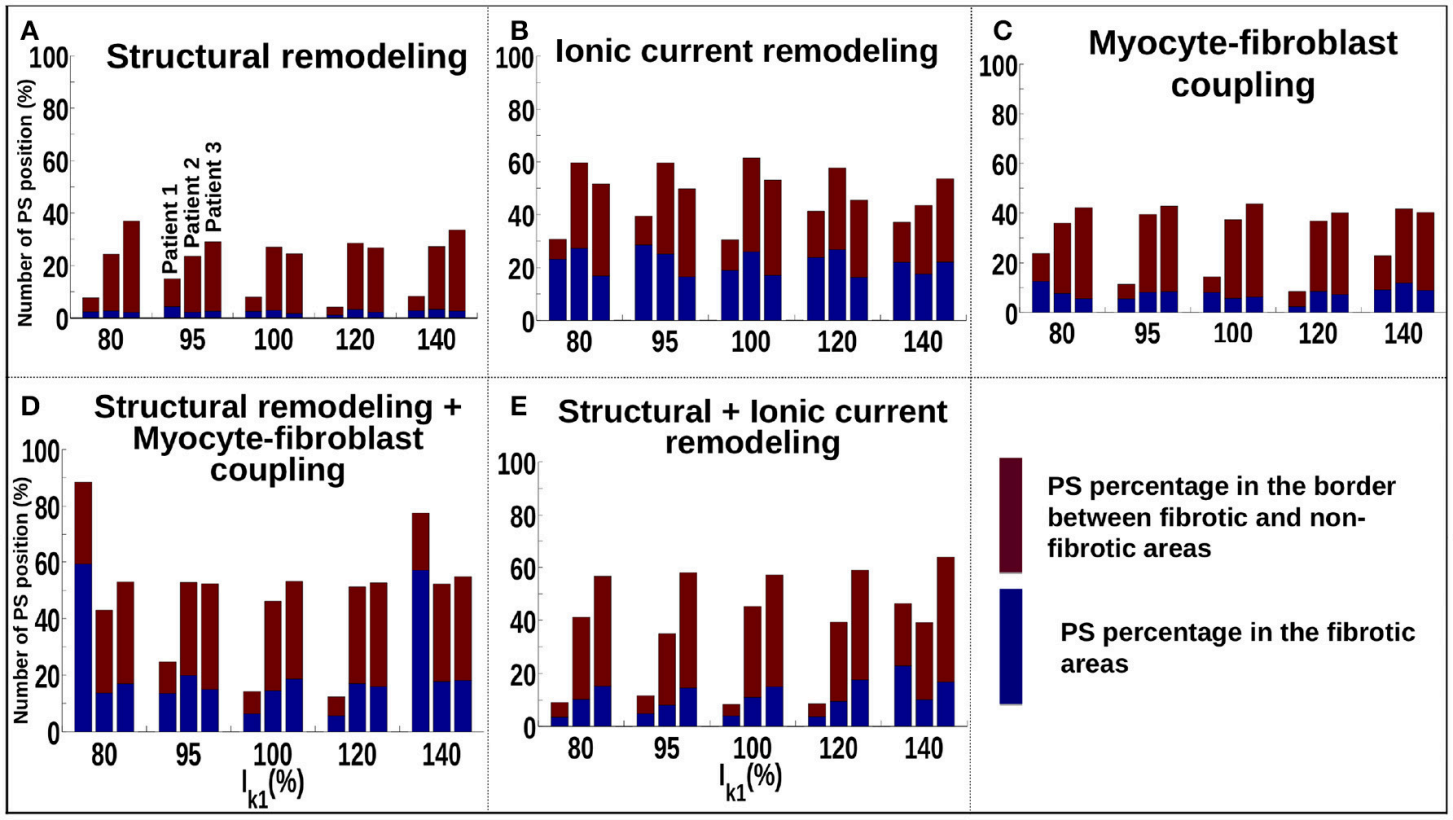
Number of PSs located in the border and in the fibrosis area for patients 1–3 for different fibrosis modeling methodologies, corresponding to: **(A)** structural remodeling, **(B)** ionic current remodeling, **(C)** myocyte-fibroblast coupling, **(D)** structural remodeling and myocyte-fibroblast coupling, **(E)** structural and ionic current remodeling.

Figure 5A shows that for the structural remodeling method, reentry cores were more likely to be located outside of the fibrotic areas than either inside the fibrotic areas or in the border regions, regardless of APD variation. PSs are also more likely to be in the fibrosis border region than in the fibrosis region itself. Figure 6A quantifies this for structural remodeling in terms of the percentages of PS positions on the border and in the fibrosis area for each patient as APD changes: more than 60% of the PS positions are located outside of the fibrotic area, regardless of the fibrosis distribution.

**Figure 7:**
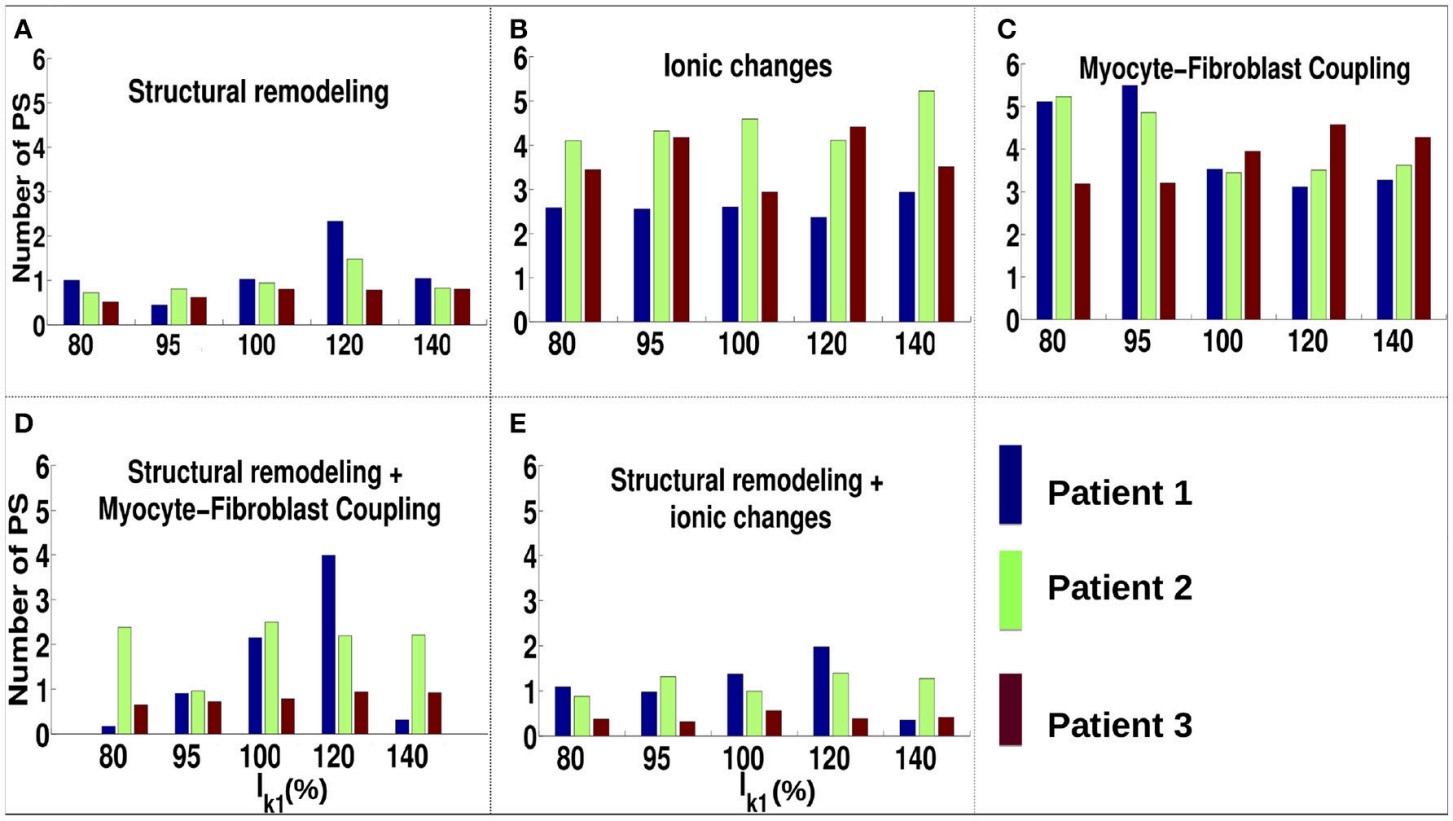
Number of PS per time unit for IK1 conductance scaled by 80–140% for different fibrosis modeling methodologies, corresponding to: **(A)** structural remodeling, **(B)** ionic current remodeling, **(C)** myocyte-fibroblast coupling, **(D)** structural remodeling and myocyte-fibroblast coupling, **(E)** structural and ionic current remodeling.

For the ionic current remodeling method (Figures 5B, 6B), PSs tend to cluster more on the border and in the fibrotic areas than using the structural remodeling methodology or the combination methodologies. With this fibrosis implementation, the number of PS per unit time in the entire atria (Figure 7B) is higher (mean ~3.53 ± 0.84 PS per unit time) than for methodologies incorporating structural remodeling (Figures 7A,D,E; mean values: ~0.95 ± 0.44, ~1.45 ± 1.02, and ~0.92 ± 0.48, respectively). The number of PS is higher and the distribution is less scattered using ionic current remodeling than using methodologies incorporating structural remodeling.

For the myocyte-fibroblast coupling method (Figures 5C, 6C), the proportion of PSs within the fibrotic area and its border is higher than the equivalent measures for structural changes, but lower than for the ionic current remodeling method. The number of PS per time unit in the entire atria (Figure 7C) using the myocyte-fibroblast coupling method is ~4.02 ± 0.80. Thus, it is higher than the number of PSs using implementations including structural remodeling. For the two combination methods (Figures 5D,E, 6D,E), PSs tend to cluster more in the border and the fibrotic areas than using the structural remodeling method alone, but the number of PS per time unit in the entire atria remains low (~1.45 ± 1.02 and ~0.92 ± 0.48 for Figures 7D,E, respectively). The myocyte-fibroblast coupling method and the ionic method result in a larger number of PSs than the structural remodeling or combination methods, and the PS density maps for these methods are more similar to each other than to the structural remodeling method (see Supplementary Figure 1). For each of the fibrosis implementation methods together with the different patient-specific fibrosis distributions, the number of PSs varies non-monotonically as the APD is decreased.

As for the control case without fibrosis, we investigated how the quantity of reentry cores located in different parts of the atria varies with APD. For each patient and each fibrosis implementation method, the variation in the percentage of PS located on all the PVs, on the LA and on the RA are presented in Supplementary Figure 2. The relative distribution of PSs in the LA compared to the RA changes between the fibrosis modeling methodologies. For example, for patient 3, there is a higher number of PSs in the RA compared to the LA for structural remodeling (~62.28 ± 10.57% of PS located on RA vs. ~30.13 ± 11.16% on LA) and also for combining structural remodeling and ionic current changes (RA ~47.24 ± 5.82% vs. LA ~37.99 ± 3.60%). Whereas, there is a higher number in the LA than the RA for the other modeling methodologies (ionic current changes: RA ~28.48 ± 5.97% vs. LA ~52.08 ± 5.48%; fibroblast coupling method alone: RA ~31.50 ± 6.75% vs. LA ~49.11 ± 8.16%; and combining structural remodeling and fibroblast coupling techniques: RA ~28.35 ± 3.45% vs. LA ~50.14 ± 5.64%).

The number of PS per unit time located on the LA, on the RA, around the FO, on the SVC and on each PV are shown in Supplementary Figure 3 for different WLs. These quantities vary with WL. Using the structural remodeling method for fibrosis, patient 1 has ~7.7, ~14.9, ~7.97, ~4.18, and ~8.29% of PS located in fibrosis areas and borders for the 5 increasing values of *I*_*K*1_, respectively (Figure 6A). Combining structural remodeling with myocyte-fibroblast coupling, the number of PS in fibrosis areas and borders are: ~88.41, ~24.69, ~14.20, ~12.34, and ~77.53%, respectively (Figure 6D), demonstrating that the effect of the fibrosis implementation formulation may be greater than the effect of the variation of WL.

## 4. Discussion

In this study, we used computer modeling to investigate the effects of WL variation on reentry in both fibrotic atria and non-fibrotic atria for three different patient-specific fibrosis distributions, with five known fibrosis implementation methods, on the same atrial mesh to exclude geometrical effects. Our study is unique in that it explores a broader range of APDs, closer to the range seen clinically, as well as investigating the effects of different AF modeling implementations. We further elucidated how structural and electrophysiological remodeling interact to determine rotor core location.

In the case of AF without fibrosis, PSs anchor on the center of the LA anterior wall and near the LA/PV junction for large APD values (Figure 3). These areas have high APD gradients (Figure 2). Decreasing the WL increases the number of PS and the distribution becomes less focused. In the case of fibrotic atria, propagation is constrained by the distribution of fibrosis through the physiological and/or structural changes made to the tissue, depending on the formulation of the implementation method. Previous clinical and computational studies have shown that tissue areas with higher fibrosis density attract more re-entrant activity than other areas (Deng et al., 2017; Cochet et al., 2018), but the trajectories can change, particularly when there are several fibrotic areas close to each other.

There is more variation in the number and location of PSs as *I*_*K*1_ varies in our simulations without fibrosis, demonstrating that the effects of WL will depend on the degree of fibrosis (comparing Figures 4, 7). The fibrosis modeling method has a large effect on the number of PSs in the fibrosis areas and its border. WL has a larger effect on rotor location for the myocyte-fibroblast coupling method and the structural remodeling method than for the other methodologies.

Incorporating atrial fibrosis in the model changed the relationship between WL and number of PSs. Figure 7 shows that there is no consistent relationship between *I*_*K*1_ conductance and the number of PSs for any of the fibrosis implementation methodologies. The number of PSs, and their locality to the fibrotic areas (Figure 6), shows a greater dependence on the fibrosis type than the *I*_*K*1_ conductance. In addition, there are large differences in the proportion of PSs located in regions of fibrosis between fibrosis implementation types. Clinically, there is evidence both for (Cochet et al., 2018) and against (Chrispin et al., 2016) the delocalization of rotational activity with high LGE-intensity. These differences may be because of variations in the type of atrial remodeling. LGE imaging does not have the resolution required to capture microscale fibrotic factors; the effects of which may change as AF progresses.

The structural remodeling method localizes less PSs in areas with high fibrosis intensity, but combining the structural remodeling method with either ionic current remodeling or fibroblast-myocyte coupling forms electrical heterogeneities that better anchor the reentrant drivers. In particular, reentry anchors around areas of fibrosis for the ionic remodeling method due to the longer APD and lower CV with this fibrosis implementation method. Similar results were found in our previous work (Roney et al., 2016), in which differences in the amounts of rotational activity and wavebreak were found between models. Zou et al. (2005) showed that AF initiation, maintenance and dynamics are strongly affected by substrate size. In addition, Panfilov (2006) suggested that the number of fibrillatory sources is proportional to the effective tissue size. In our study, the low presence of PS locations in high fibrosis density zones when modeling structural changes alone may be explained by the large quantity of elements that are removed in these regions, making the effective size of the tissue per unit surface area too small for rotor creation. The effective tissue size per surface unit is also low for cases with structural remodeling combined with ionic remodeling or fibroblast coupling, leading to a low presence of PS locations in high fibrosis density regions. Incorporating ionic remodeling or fibroblast coupling alone to implement fibrosis does not affect the effective tissue size per unit area, and results in a larger quantity of PSs. The higher number of PSs obtained with the fibroblast coupling method compared to the ionic remodeling method may be explained by the way in which the gradient of the physiological parameters is implemented for the two methods. The ionic remodeling method imposes an abrupt decrease in the conductivity at the border between fibrotic and non-fibrotic zones, whereas the fibroblast coupling method has five different levels of conductivity, changing from non-fibrotic to the highly fibrotic zones. This suggests that a more gradual spatial change in conductivity may lead to a larger number of PSs.

Deng et al. (2017) investigated the sensitivity of rotor location to variations in APD and CV, for simulations incorporating fibrosis modeled with modified ionic and conductivity properties. In their simulations, rotors were overwhelmingly located in border regions between fibrotic and healthy tissue regardless of APD and CV values; however, the specific border regions that contained rotors varied depending on the APD and CV parameters. A subset of rotor locations in the modified APD and CV cases were also present in the baseline case (35–80% agreement). The ionic remodeling fibrosis modeling methodology in our study is similar to the methodology used by Deng et al. (2017). We found a higher proportion of PSs in the fibrosis area and its border using this methodology than either the structural remodeling or fibroblast coupling method, and this proportion was high for all *I*_*K*1_ values. For the ionic fibrosis implementation, the number and distribution of PSs was similar for the different *I*_*K*1_ values. As such, our findings for the ionic fibrosis implementation agree with those of Deng et al. (2017) since we also see PSs in the fibrosis border zone, with some sites preserved as well as some different sites emerging as *I*_*K*1_ is varied.

However, in our simulations, there were also a significant proportion of PSs outside of the fibrosis area and border region, particularly around areas with significant APD gradient - for example the LA/PV junction. PS locations were found in areas of long APD (Figure 3), which is consistent with previous studies showing that areas of long APD attract PSs more strongly than inexcitable obstacles (Calvo et al., 2014; Defauw et al., 2014). Our model includes regional heterogeneity between atrial regions, resulting in APD heterogeneities in addition to those from the fibrosis distribution. Further differences in behavior may be attributable to model differences which include our representation of a bilayer mesh with discrete interatrial connections as opposed to a continuous three-dimensional (3D) mesh.

The parameters used for the fibrosis modeling methodologies will affect PS distributions. For the ionic current remodeling method, a threshold intensity must be chosen to assign fibrotic regions, while for the myocyte-fibroblast coupling method, the number of fibroblasts must be chosen. The ionic and fibroblast methods include different degrees of conductivity changes. Matching simulation results with clinical data depends on these parameters. Parameter choice sensitivity was investigated in previous studies (Morgan et al., 2016; Roney et al., 2016; Zahid et al., 2016).

Catheter ablation therapy typically focuses on the LA. Our results show that the proportion of PSs in the RA is typically low for longer APD values; however, this increases as APD decreases (see Figure 4A). This suggests that for shorter WLs, the RA may play a larger role in driving the arrhythmia and RA ablation may be required. In addition, for the control case without fibrosis, the number of PSs and arrhythmia complexity increases as WL is decreased (see Figure 4B). Thus, estimating WL (as the product of AF cycle length and CV) during a procedure may help with therapy planning.

Multiple studies have investigated the effects of ERP, CV and tissue area on the probability that AF sustains. For example, Ravelli and Allessie (1997) found that ERP shortening induced by atrial stretch increased AF vulnerability; Zhuang et al. (2011) report that AF recurrence is more likely in dilated atria because of the increase in size; and Lee et al. (2013) found that both ERP and area affect the probability that AF sustains. Rensma et al. (1988) suggested that ERP and CV alone are poor predictors of susceptibility to reentry, and that the combined measure of WL is a better predictor. Reentry occurs when the path length taken by the wavefront is longer than the WL. WL changes as AF progresses due to increased electrical and structural remodeling. AF susceptibility can then be considered in terms of a critical WL value (Jacquemet et al., 2005), or a fibrillation number, which combines the atrial area and WL into a single measure that is higher in patients with post PV isolation AF inducibility (Hwang et al., 2015). In our study, the number of PSs increases with decreasing WL for simulations without fibrosis, meaning that shorter WL AF simulations are more likely to sustain. However, this was not the case for simulations with fibrosis. Fareh et al. (1998) found that ERP heterogeneity was more important than WL in predicting AF susceptibility, which may be the case for our simulations with fibrosis in which the presence of fibrosis results in conduction and depolarization gradients.

Reentry dynamics depend on both depolarization gradients, with increased PS density in regions of high APD gradient (for example the LA/PV junction), and fibrosis distribution. PS density maps show a closer correspondence with LGE-MRI intensity and are less affected by WL for ionic remodeling and myocyte-fibroblast coupling than for structural remodeling. The effects of fibrosis may outweigh the effects of WL variation in later stages of AF when the fibrosis burden is higher. During the onset of AF, WL and electrophysiology may be more important since electrical remodeling typically occurs before substrate remodeling.

## 5. Limitations

To investigate the effects of WL and fibrosis in isolation, we used the same atrial geometry for all simulations. However, patient-specific geometry and volume will affect AF dynamics (Cochet et al., 2018). The choice of human atrial cell model affects PS locations (Cherry and Evans, 2008; Wilhelms et al., 2013), but we did not investigate this. We changed WL through variations in ERP, but CV will also affect WL and reentry dynamics (Lim et al., 2017). In addition, we use a 2D bilayer model of the atria, rather than a full 3D model, so we cannot account for the effects of atrial thickness on rotor dynamics (Dutta et al., 2016). We used identical fibrosis distributions for the endocardial and epicardial surfaces of the LA and only included fibrosis on the RA epicardium; however, fibrosis is known to occur in the pectinate muscles (Spach et al., 1988) and the development of atrial fibrosis varies across the atrial thickness (Verheule et al., 2014), starting in the epicardial layer. We normalized LGE intensity to the maximum value for each patient, rather than working with either a number of standard deviations above the blood pool mean or the image intensity ratio for defining fibrotic tissue, which means that the amount of fibrosis included in each model does not necessarily reflect the Utah score for the patient. The choice of LGE-MRI intensity threshold used to define fibrotic and non-fibrotic areas in the image processing and modeling pipeline will affect the resulting AF dynamics, but the optimum value is unknown. For the fibroblast-myocyte coupling model of fibrosis, the fibroblast-myocyte gap-junction conductance value remains experimentally uncertain; *in vivo* measurements are required to validate fibroblast-myocyte coupling properties used in the simulations.

## 6. Conclusion

Cardiac WL affects reentry dynamics. Without fibrotic remodeling, increasing the cardiac WL decreased arrhythmia complexity, such that reentrant activities anchored and the number of PSs reduced. WL variation also modified reentry dynamics in fibrotic atria; however, this relationship is more complicated, as it also depends on the fibrosis distribution and fibrosis implementation method. The effects of WL, electrophysiology and APD heterogeneity should be included in computational simulations of AF. With higher fibrosis content, simulated PSs are less affected by WL and more likely to be found close to fibrotic patches. This has implications for model personalization as it suggests that it is important to tune WL for patients with a low degree of fibrotic remodeling, whilst LGE maps alone may be sufficient to predict behavior for patients with significant fibrosis, for which fibrosis implementation method is important.

## Author contributions

MS ran the computational simulations, analyzed data and generated graphs. CR and EV conceived and designed the study and wrote analysis tools for the project. MS, CR, and EV drafted the manuscript. JB provided technical input on the simulation tools. MM and RD analyzed the CardioInsight clinical data. HC segmented the LGE-MRI data.

### Conflict of interest statement

The authors declare that the research was conducted in the absence of any commercial or financial relationships that could be construed as a potential conflict of interest.
