# Supplementary material for: Wavelength and Fibrosis Affect Phase Singularity Locations During Atrial Fibrillation
Source: Front Physiol. 2018 Sep 10;9:1207. doi: 10.3389/fphys.2018.01207 (PMC6139329; doi:10.3389/fphys.2018.01207)
Supplement: Supplementary file 1 [file Data_Sheet_1.PDF]

Supplementary material:

Wavelength and Fibrosis Affect Phase Singularity Locations During Atrial Fibrillation

Mirabeau Saha, Caroline Roney, Jason Bayer, Marianna Meo,
Hubert Cochet, Remi Dubois, and Edward Vigmond

July 3, 2018

Supplementary figures

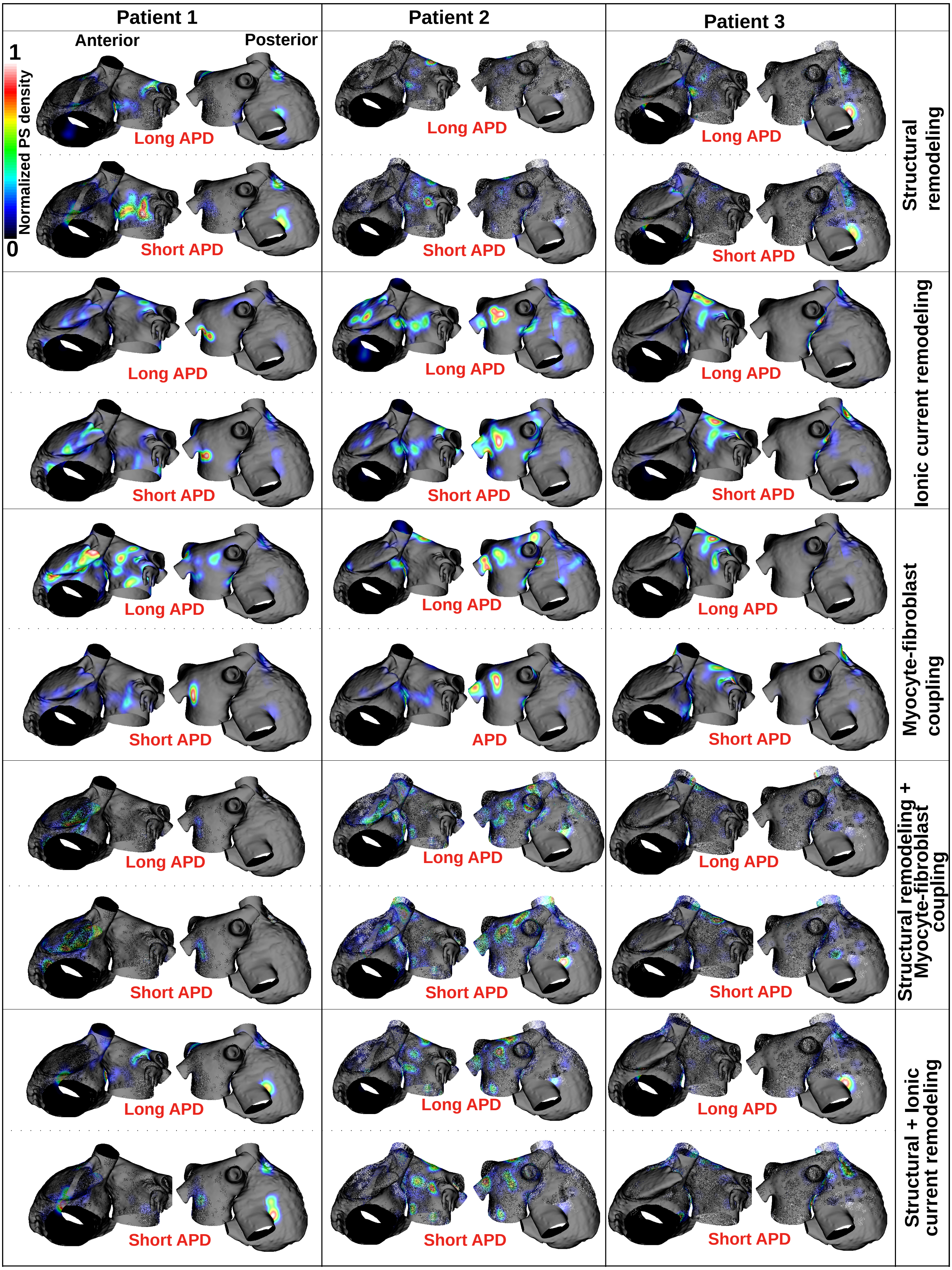

Figure 1: Normalised phase singularity (PS) density maps for I_{K1} conductance scaled by 80% to 140% of the control conductance value.

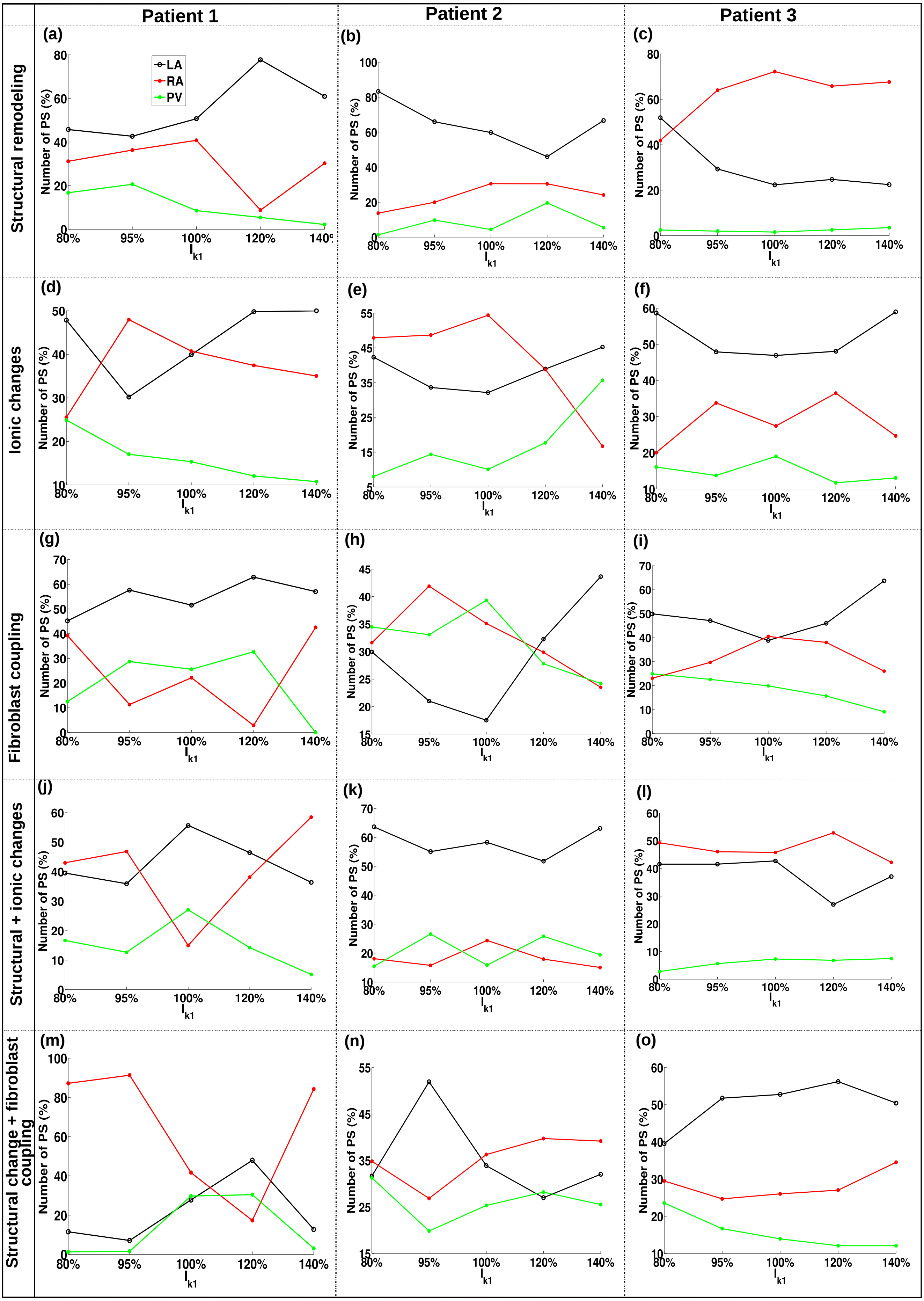

Figure 2: Change in the distribution of PS on the main subdivisions of the atria (all PVs, LA body (excluding PVs) and RA) as a function of I_{K1} conductance.

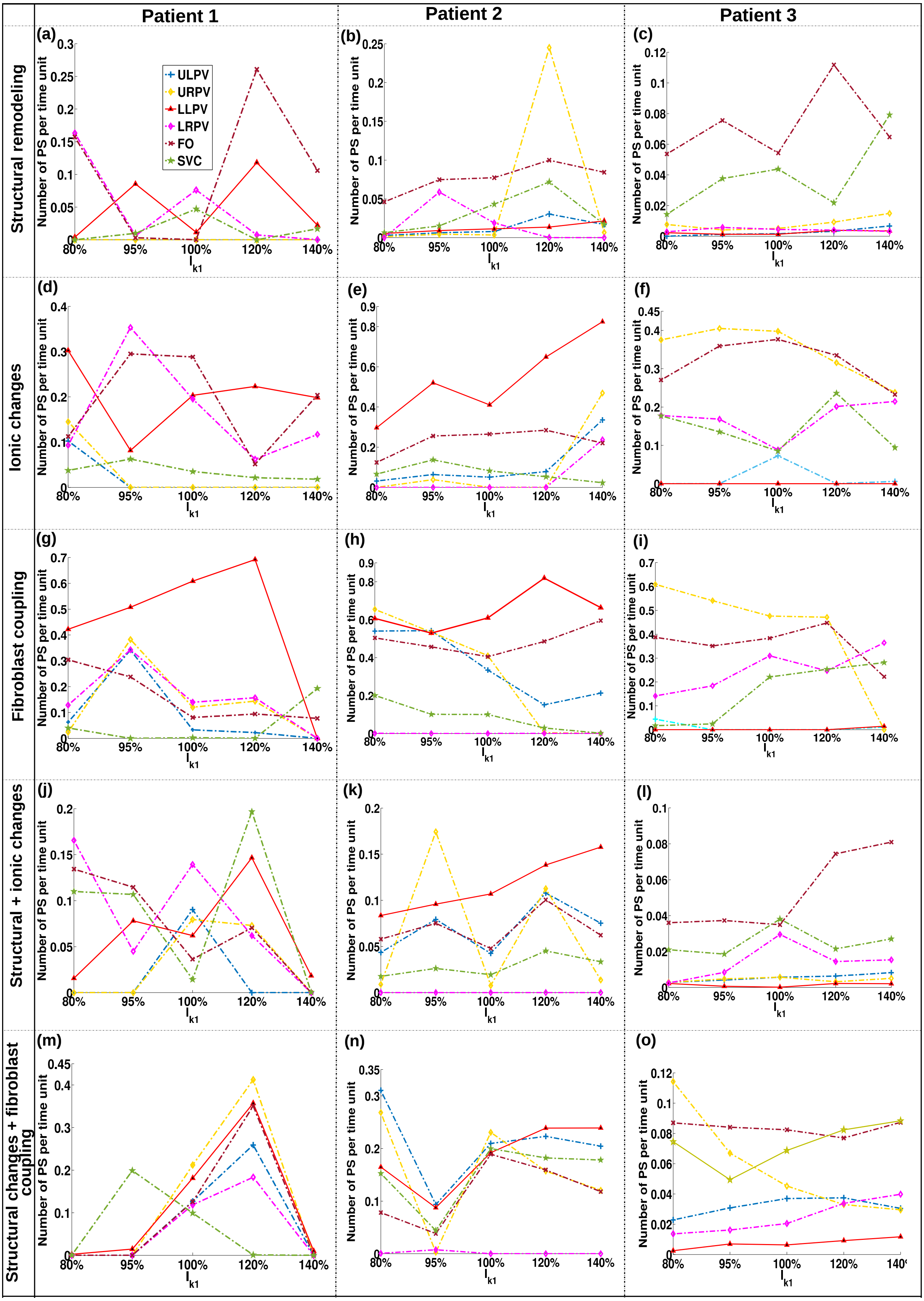

Figure 3: Number of PS per time unit in atrial regions as a function of I_{K1} conductance.

PS density correlation matrices

1.0	0.39443	0.62942	0.37475	0.5425
0.39443	1.0	0.55305	0.24231	0.5351
0.62942	0.55305	1.0	0.2355	0.4739
0.37475	0.24231	0.2355	1.0	0.41308
0.5425	0.5351	0.4739	0.41308	1.0

Structural remodelling

1.0	0.63494	0.68699	0.74141	0.70226
0.63494	1.0	0.88467	0.67343	0.67685
0.68699	0.88467	1.0	0.73589	0.76931
0.74141	0.67343	0.73589	1.0	0.90801
0.70226	0.67685	0.76931	0.90801	1.0

Ionic remodelling

1.0	0.56515	0.57283	0.29581	0.45999
0.56515	1.0	0.68161	0.43481	0.3458
0.57283	0.68161	1.0	0.36977	0.4117
0.29581	0.43481	0.36977	1.0	0.26808
0.45999	0.3458	0.4117	0.26808	1.0

Fibroblast coupling

1.0	0.34361	0.29266	0.20245	0.88576
0.34361	1.0	0.52657	0.20498	0.37396
0.29266	0.52657	1.0	0.61165	0.30191
0.20245	0.20498	0.61165	1.0	0.22279
0.88576	0.37396	0.30191	0.22279	1.0

Structural remodelling + Fibroblast coupling

1.0	0.70537	0.43862	0.61352	0.39286
0.70537	1.0	0.30391	0.61356	0.45701
0.43862	0.30391	1.0	0.49682	0.38654
0.61352	0.61356	0.49682	1.0	0.32877
0.39286	0.45701	0.38654	0.32877	1.0

Structural + Ionic remodelling

Table 1: **Patient 1 PS density correlation matrices.** Each matrix shows the correlation of PS density maps for I_{K1} conductance scaled by 80, 90, 100, 120, 140%.

1.0	0.75461	0.7707	0.29698	0.626
0.75461	1.0	0.86209	0.37215	0.58642
0.7707	0.86209	1.0	0.45447	0.60269
0.29698	0.37215	0.45447	1.0	0.62614
0.626	0.58642	0.60269	0.62614	1.0

Structural remodelling

1.0	0.84978	0.88689	0.85831	0.64729
0.84978	1.0	0.91724	0.89801	0.67246
0.88689	0.91724	1.0	0.88377	0.64501
0.85831	0.89801	0.88377	1.0	0.73872
0.64729	0.67246	0.64501	0.73872	1.0

Ionic remodelling

1.0	0.7662	0.64494	0.68556	0.61294
0.7662	1.0	0.65853	0.68516	0.54936
0.64494	0.65853	1.0	0.6564	0.46123
0.68556	0.68516	0.6564	1.0	0.71503
0.61294	0.54936	0.46123	0.71503	1.0

Fibroblast coupling

1.0	0.51702	0.80652	0.71107	0.6856
0.51702	1.0	0.626	0.66388	0.64818
0.80652	0.626	1.0	0.85375	0.80234
0.71107	0.66388	0.85375	1.0	0.91951
0.6856	0.64818	0.80234	0.91951	1.0

Structural remodeling + Fibroblast coupling

1.0	0.75716	0.92418	0.7794	0.81059
0.75716	1.0	0.71271	0.89612	0.69804
0.92418	0.71271	1.0	0.76999	0.79351
0.7794	0.89612	0.76999	1.0	0.76365
0.81059	0.69804	0.79351	0.76365	1.0

Structural + Ionic remodelling

Table 2: **Patient 2 PS density correlation matrices**

1.0	0.4213	0.50913	0.4019	0.50458
0.4213	1.0	0.68589	0.59208	0.48969
0.50913	0.68589	1.0	0.6608	0.59191
0.4019	0.59208	0.6608	1.0	0.67305
0.50458	0.48969	0.59191	0.67305	1.0

Structural remodelling

1.0	0.91337	0.65876	0.84805	0.88686
0.91337	1.0	0.69225	0.89383	0.9006
0.65876	0.69225	1.0	0.70273	0.69391
0.84805	0.89383	0.70273	1.0	0.88057
0.88686	0.9006	0.69391	0.88057	1.0

Ionic remodelling

1.0	0.78803	0.69597	0.75905	0.58326
0.78803	1.0	0.80287	0.84687	0.66523
0.69597	0.80287	1.0	0.91006	0.59738
0.75905	0.84687	0.91006	1.0	0.68937
0.58326	0.66523	0.59738	0.68937	1.0

Fibroblast coupling

1.0	0.77305	0.7437	0.69557	0.70207
0.77305	1.0	0.95758	0.81752	0.83502
0.7437	0.95758	1.0	0.84181	0.8351
0.69557	0.81752	0.84181	1.0	0.93573
0.70207	0.83502	0.8351	0.93573	1.0

Structural remodeling + Fibroblast coupling

1.0	0.64802	0.82542	0.55254	0.56147
0.64802	1.0	0.56638	0.89029	0.83668
0.82542	0.56638	1.0	0.48146	0.47695
0.55254	0.89029	0.48146	1.0	0.88534
0.56147	0.83668	0.47695	0.88534	1.0

Structural + Ionic remodelling

Table 3: **Patient 3 PS density correlation matrices**
